# Organocatalytic C–H activation reactions

**DOI:** 10.3762/bjoc.8.159

**Published:** 2012-08-27

**Authors:** Subhas Chandra Pan

**Affiliations:** 1 Department of Chemistry, Indian Institute of Technology Guwahati, North Guwahati, Assam, 781039, India

**Keywords:** asymmetric, C–H activation, non-asymmetric, organocatalysis, organocatalytic

## Abstract

Organocatalytic C–H activation reactions have recently been developed besides the traditional metal-catalysed C–H activation reactions. The recent non-asymmetric and asymmetric C–H activation reactions mediated by organocatalysts are discussed in this review.

## Introduction

C–H activation reactions have recently been found to be a powerful method for the formation of C–C and C–X bonds [1–3]. The advantage of this method is that it does not require the functional group of the carbon atom, as in the conventional approach. Transition-metal-catalysed C–H bond functionalization reactions have been well-studied and different site-selective (regioselective and chemoselective) reactions have been reported [1–3]. However, one drawback of this approach is the requirement of the removal of the metal impurity from the products. An organocatalytic approach is attractive in this sense as it is metal-free, cost-effective, and favoured by the pharmaceutical industry for being one of the “key green chemistry research areas” [4–6]. This review describes the current “state of the art” in organocatalyzed C–H activation reactions and highlights recent advances in sp^2^ and sp^3^ C–H bond functionalization. For simplicity, iodide or hypervalent iodine-mediated metal-free C–H transformations will not be covered in this review.

## Review

### Organocatalytic sp^3^ C–H bond activation reactions

#### Non-asymmetric variants

**Scheme 1:**
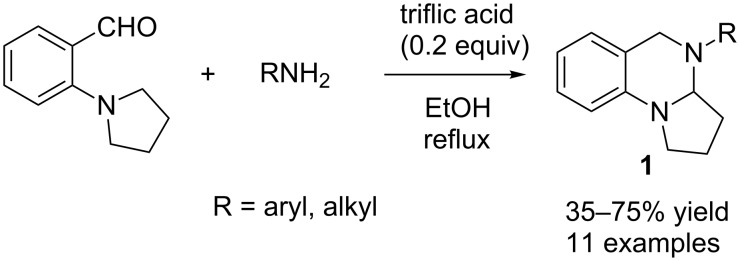
Triflic acid-catalysed synthesis of cyclic aminals.

**Scheme 2:**
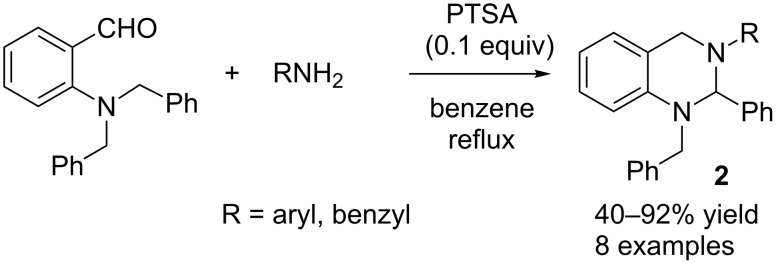
PTSA-catalysed synthesis of cyclic aminals.

*tert-*Amino effect: The “*tert*-amino effect” refers to the ring-closure reactions that proceed by redox processes for C–C and C–X bond formation within conjugated systems [7–9]. One well-known example of the “*tert*-amino effect” is the cyclization of *N*,*N*-dialkyl-substituted anilines with imines to generate cyclic aminals. The groups of Seidel and Akiyama independently reported mild organocatalytic approaches to cyclic aminals involving aminobenzaldehydes and primary amines [10–11]. Previously, only preformed imines were used for this purpose, and the reactions were mostly thermally controlled [12–15]. Seidel and co-workers found that the combination of triflic acid and ethanol provided an effective system for the formation of cyclic aminals (Scheme 1), whereas the combination of *para*-toluenesulfonic acid (PTSA) and benzene was the optimum system for Akiyama and co-workers (Scheme 2).

After evaluating different Lewis and Brønsted acids with their equivalents as well as different solvents, Seidel and co-workers found that 0.2 equivalents of triflic acid in ethanol under reflux provided the maximum yield of the desired product **1**. The reaction was examined with different amines and moderate to good yields (35–75%) were obtained (Scheme 1) [10]. In certain cases, a stoichiometric amount of triflic acid was required to obtain acceptable yields of the desired products.

**Scheme 3:**
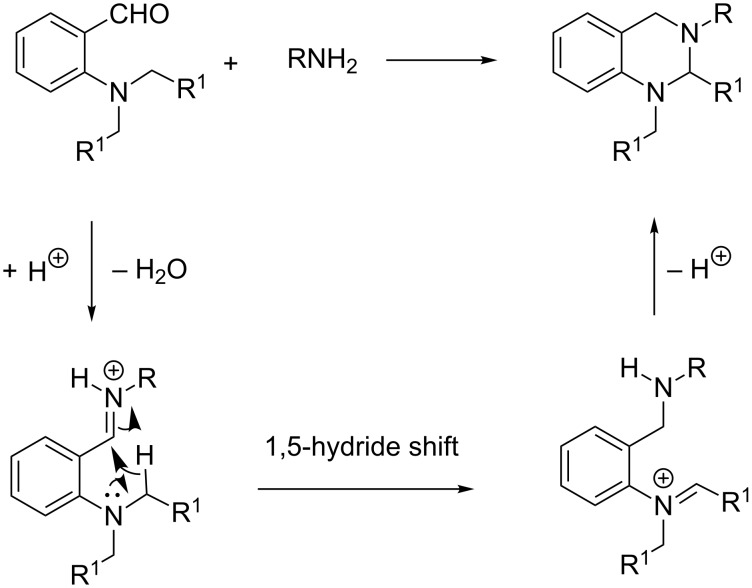
Plausible mechanism for cyclic aminal synthesis.

Akiyama and co-workers screened different Brønsted acid catalysts for their reaction, with PTSA emerging as the most effective catalyst [11]. Employing an optimized set of conditions, the reaction was conducted with different amines, and good to excellent yields (40–92%) were obtained (Scheme 2). A plausible mechanism for these reactions is depicted in Scheme 3.

After initial imine formation, a Brønsted acid promoted 1,5-hydride shift occurs yielding an imino-amine intermediate. Cyclization of the newly formed amine affords the desired aminal product.

**Scheme 4:**
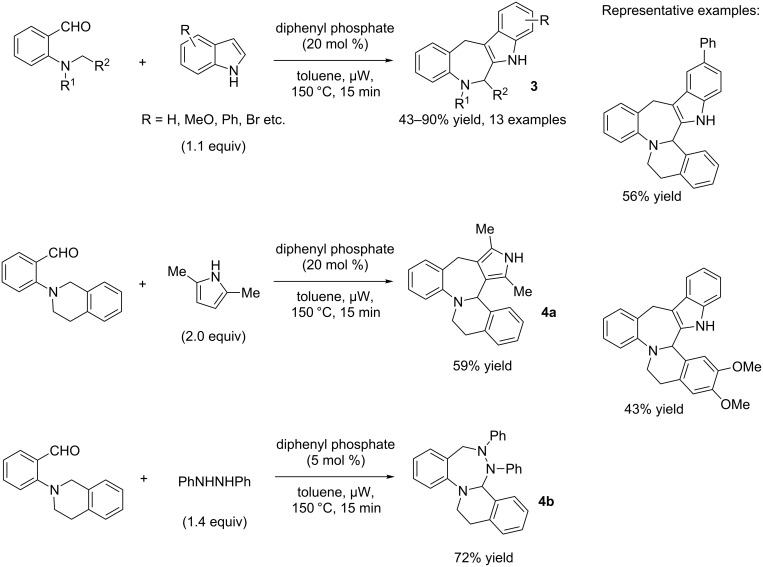
Annulation cascade reaction with double nucleophiles.

In 2011, Seidel and co-workers reported an indole-annulation cascade reaction with *N*,*N*-(dialkylamino)benzaldehydes, a process in which a 1,5-hydride shift occurs followed by larger ring formation [16]. After the evaluation of different Brønsted acid catalysts, diphenylphosphate (20 mol %) was identified as a suitable catalyst for the reaction. Toluene again was the solvent of choice, and microwave irradiation was used for a shorter reaction time. The scope of the reactions was broad allowing different indoles and a variety of *N*,*N*-(dialkylamino)benzaldehydes to be employed, and the products **3** were obtained in good to excellent yields (Scheme 4) [16].

**Scheme 5:**
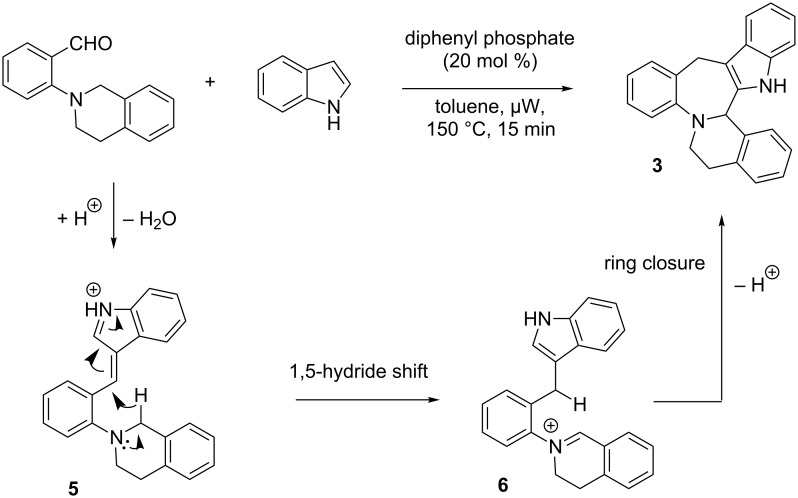
Mechanism for the indole-annulation cascade reaction.

For some substrates, 50 mol % of the catalyst was required to obtain reasonable yields of the products. The authors also successfully extended this methodology to other double nucleophiles, i.e., 2,5-dimethylpyrroles and *N*,*N*’-diphenylhydrazine, and the products **4a** and **4b** were isolated in good yields (Scheme 4). A plausible mechanism was suggested by the authors and is shown in Scheme 5.

Initially, the vinylogous iminium (azafulvenium) ion **5** is formed from the acid-catalyzed dehydration reaction of tertiary aminobenzaldehyde and indole (Scheme 5). Subsequently, **5** undergoes a 1,5-hydride shift to generate iminium ion **6**. Finally, ring closure and proton loss provides the formation of the product **3**.

#### Redox alkylation

**Scheme 6:**
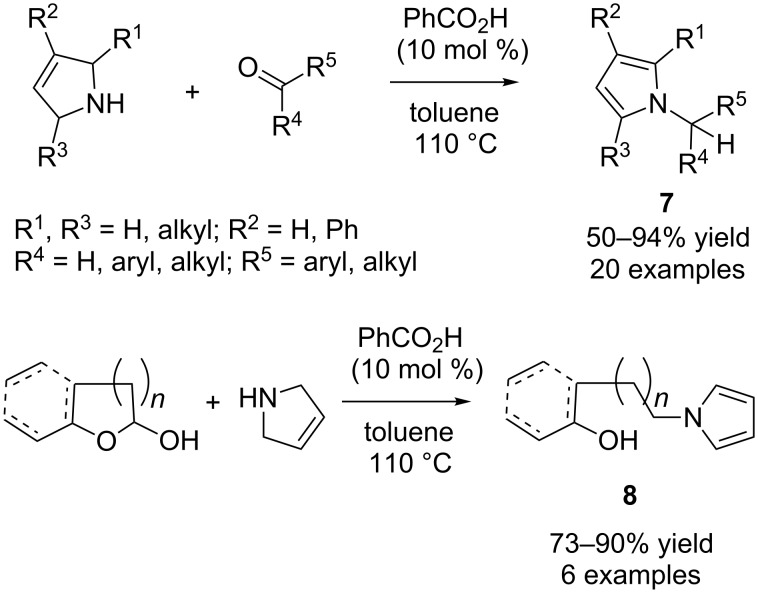
Synthesis of *N*-alkylpyrroles and δ*-*hydroxypyrroles.

In 2009, Tunge and co-workers demonstrated the synthesis of *N*-alkyl-pyrroles by redox isomerisation from the reaction of 3-pyrroline and aldehydes or ketones (Scheme 6) [17].

A series of Brønsted acids as catalysts was screened for this reaction, and the best reaction efficiency in terms of yield and reaction time was achieved with benzoic acid (10 mol %). The scope of the reaction was investigated and was found to tolerate a wide variety of functional groups including nitro, nitriles, ether and acetals delivering the products **7** in good to excellent yields (50–94%). The reaction was also compatible with different ketones although extended reaction times were required to obtain good yields of the desired products. Interestingly, when the substrates were extended to five and six-membered lactols, δ*-*hydroxypyrroles **8** were achieved as the products in good yields (Scheme 6).

**Scheme 7:**
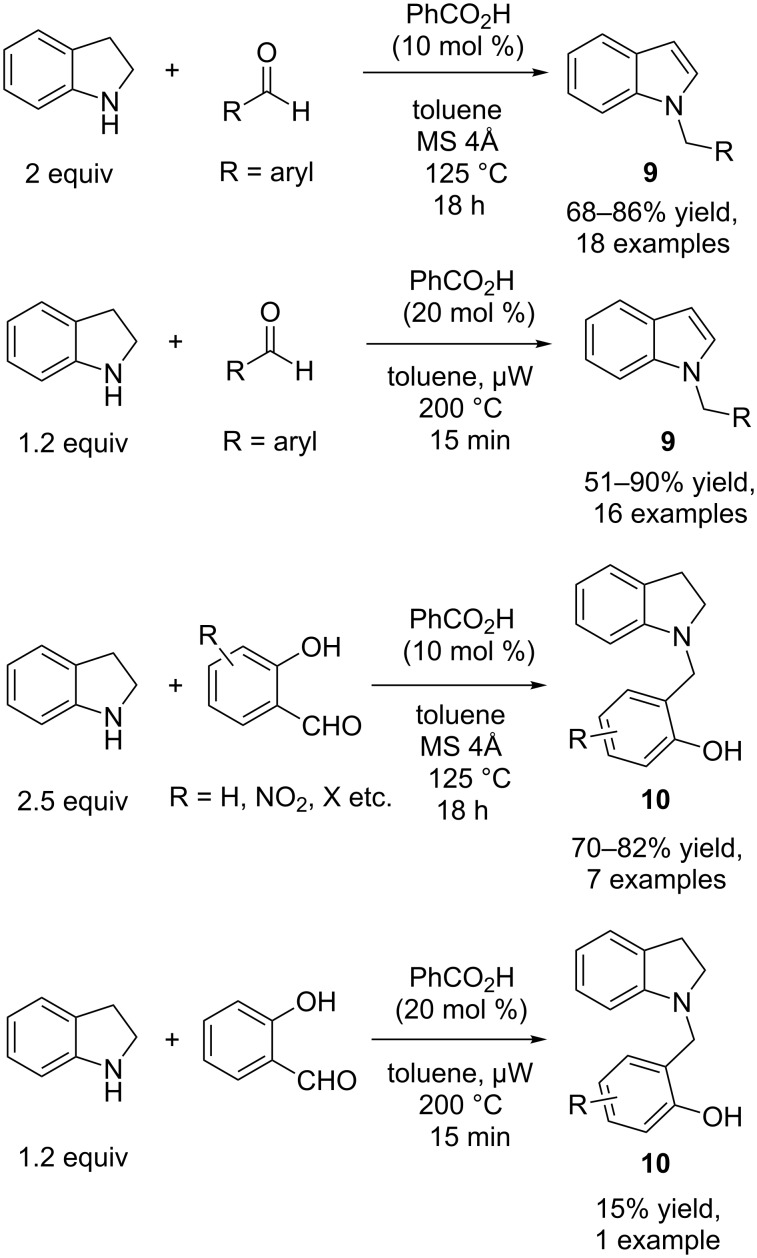
Synthesis of *N*-alkylindoles **9** and *N*-alkylindolines **10**.

Later, Pan and Seidel independently extended this methodology to indolines using benzoic acid as the catalyst, conducting the reaction under reflux and microwave irradiation conditions, respectively, to generate indole **9** (Scheme 7) [18–19].

Only aryl aldehydes are able to perform the reaction, but the yields as reported by both groups are very good. Besides the usual reaction between indolines and aldehydes, both groups also found that intermolecular hydride transfer occurred when salicylaldehyde was employed as the substrate, and the *N*-alkyl-indoline product **10** was obtained in good yields (70–82%) mainly by the method of the Pan group (Scheme 7). In this case, another molecule of indoline acts as the hydride donor and is converted to indole.

**Scheme 8:**
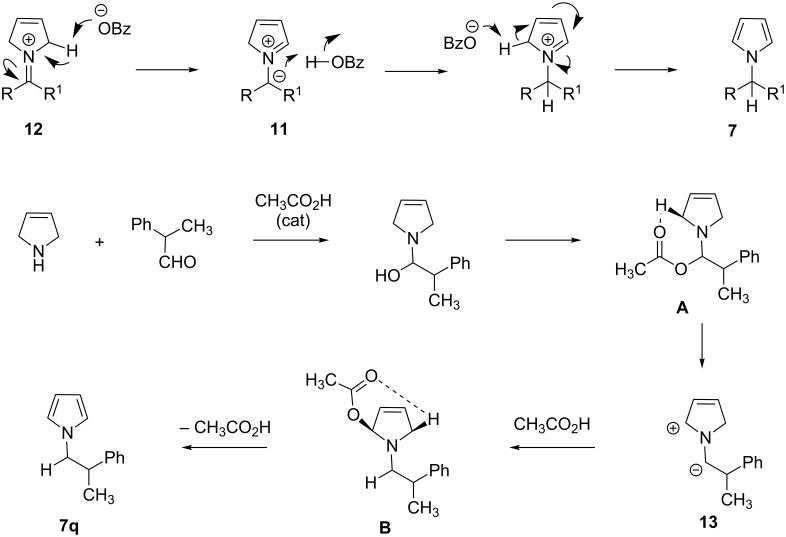
Mechanistic study for the *N*-alkylpyrrole formation.

Both Tunge and Pan suggested redox isomerization in the formation of their products, but did not provide a detailed mechanism. 1,3-Hydride shift could be the most direct pathway for the formation of the redox isomerization products. However, Seidel pointed out that a 1,3-hydride shift will occur antarafacially and is geometry-forbidden. An alternative explanation is the formation of azomethine ylide intermediate **11** (Scheme 8) [19–20].

**Scheme 9:**
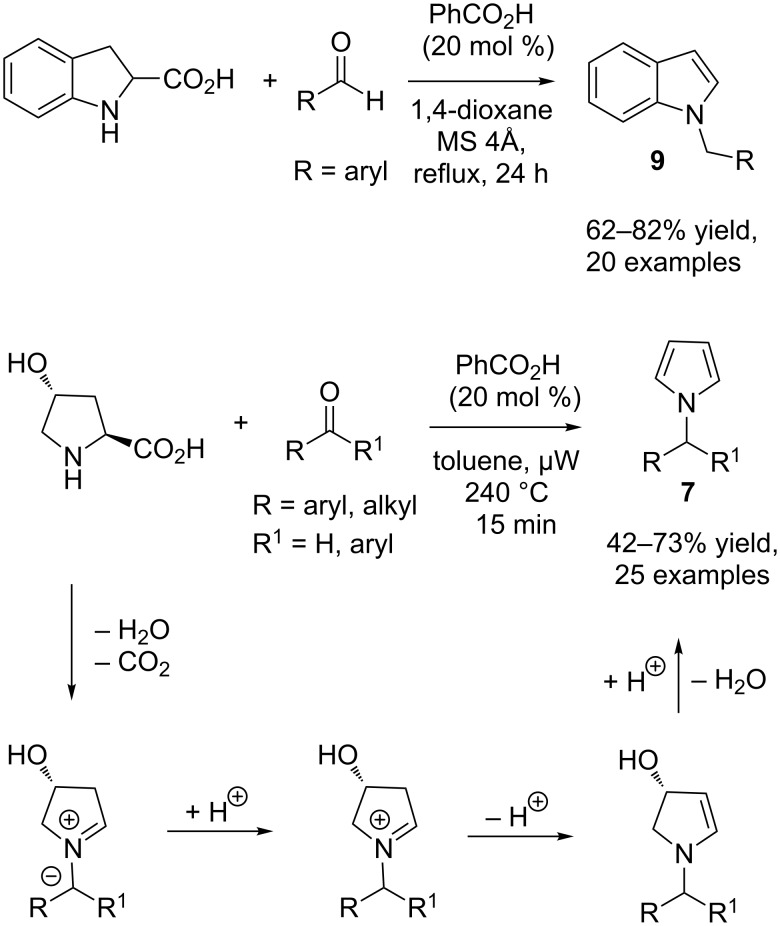
Benzoic acid catalysed decarboxylative redox amination.

The carbanion of ylide **11** is then protonated by benzoic acid, and the resulting benzoate anion supports the aromatization process. In fact, Seidel and co-workers provided the experimental evidence for the existence of azomethine ylide intermediates in the Tunge pyrrole formation and in the formation of *N*-alkylindoles from indoline [19]. These reactions are considered C–H activation reactions, as during the azomethine ylide formation, the C–H bond that is cleaved is not activated by electron-withdrawing (such as ester) groups. Recently, Xue, Cheng and co-workers carried out detailed DFT and MP2 computational studies for the reaction of 3-pyrroline and 2-phenylpropanal using acetic acid as the catalyst [21]. Interestingly, the authors could not find the existence of free iminium ion **12** in the rearrangement. They indicated that the formation of acetic acid assisted azomethine ylide **13** is the most plausible pathway for the rearrangement process [21]. The first step is the nucleophilic addition of an amine to the carbonyl group to generate a carbinolamine intermediate (Scheme 8). It then becomes *O*-acetylated by acetic acid to form intermediate **A**. Azomethine ylide **13** is then produced by extrusion of acetic acid from intermediate **A**. Protonation of **13** generates another *O*-acetyl intermediate **B**, and finally, regeneration of acetic acid and aromatization provides the pyrrole product **7q**. Pan and Seidel also independently disclosed examples of Brønsted acid catalysed decarboxylative redox-amination reactions. 2-Carboxyindoline and *trans*-4-hydroxyproline were used as the substrates, respectively [22–23]. Benzoic acid as catalyst and 1,4-dioxane as solvent was identified by the Pan group as the best system for the reaction (Scheme 9) [22].

Different aromatic and heteroaromatic aldehydes were screened and *N*-alkylindole products **9** were isolated in moderate to good yields (62–82%). One current limitation of this method is its restriction to non-enolisable aldehydes. In contrast, Seidel and co-workers successfully applied both aromatic and enolisable aldehydes and ketones to their reaction and the desired *N*-alkylpyrrole products **7** were formed in moderate to good yields (42–73%) under microwave irradiation [23]. Both groups suggested azomethine ylide as the intermediate in their reactions (Scheme 9).

#### Asymmetric variants

**Scheme 10:**
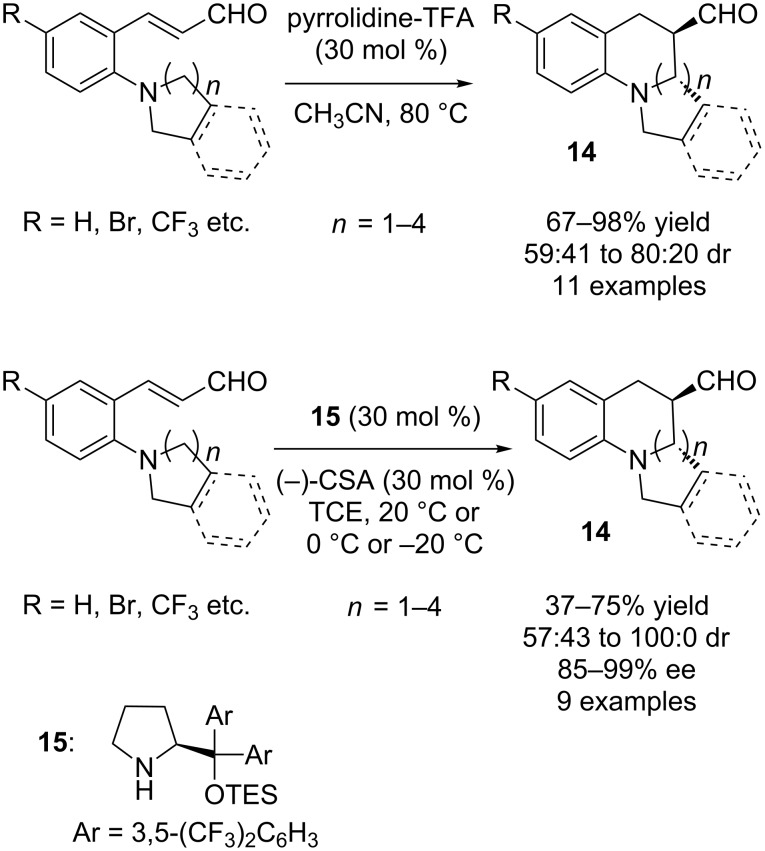
Organocatalytic redox reaction of *ortho*-(dialkylamino)cinnamaldehydes.

The first organocatalytic asymmetric C–H activation reaction was disclosed by Kim and co-workers for the synthesis of chiral tetrahydroquinolines **14** (Scheme 10) [24].

**Scheme 11:**
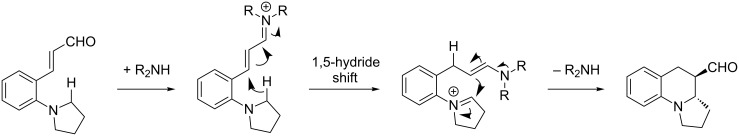
Mechanism for aminocatalytic redox reaction of *ortho*-(dialkylamino)cinnamaldehydes.

*ortho*-(Dialkylamino)cinnamaldehydes were employed as the substrates for this aminocatalytic intramolecular redox reaction. The authors first demonstrated a non-asymmetric version of the reaction using pyrrolidine-TFA as catalyst in acetonitrile. High yields (67–98%) and moderate to good diastereoselectivities (59:41 to 80:20) were obtained for amine donors of different ring size [24]. After successfully performing the non-asymmetric synthesis of tetrahydroquinolines by C–H activation, the authors embarked on the asymmetric transformation utilizing chiral secondary amine catalysis. After the screening of different chiral amine catalysts, solvents, and acid additives, as well as temperatures, chiral pyrrolidine catalyst **15** in combination with (−)-camphorsulfonic acid (CSA) in 1,1,2-trichloroethane (TCE) at 20 °C provided the desired product in highest enantioselectivity (89% ee). Under the optimized conditions, a range of *ortho-*(dialkylamino)cinnamaldehydes were employed and chiral tetrahydroquinoline products **14** were obtained in moderate to good yields (37–75%), moderate to excellent diastereoselectivities (57:43 to 100:0 dr) and high to excellent enantioselectivities (85–99% ee) (Scheme 10) [24]. For some substrates, the reaction temperature was lowered to 0 or −20 °C in order to attain high enantioselectivities. A possible mechanism for the transformation is shown in Scheme 11.

At first, the secondary amine catalyst reacts with the α,β-unsaturated aldehyde to generate an iminium ion. Subsequent 1,5-hydride shift generates the corresponding enamine. Finally, Mannich-type cyclization provides the product **14**, and the secondary amine catalyst is regenerated (Scheme 11).

**Scheme 12:**
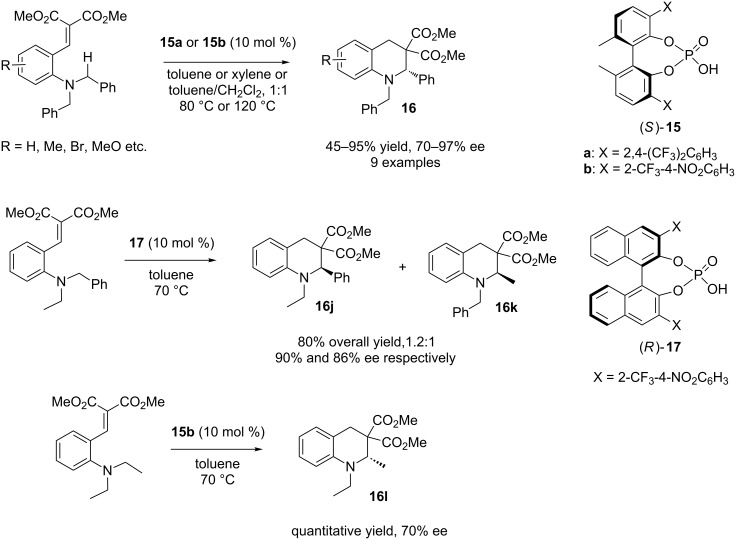
Asymmetric synthesis of tetrahydroquinolines having *gem*-methyl ester groups.

The following year, Akiyama and co-workers reported another organocatalytic asymmetric synthesis of tetrahydroquinolines using chiral phosphoric acid as the catalyst [25]. In this instance, benzylidene malonates were used as the hydride acceptor. Another important feature of this report by the Akiyama group is the predominant use of *N,N*-dibenzylamine as the amine donor in their reaction instead of cyclic tertiary amines as used by the Kim group. The present authors employed biphenyl-based chiral phosphoric acid catalysts **15a** and **15b** and moderate to high yields (45–95%) and excellent enantioselectivities (70–97% ee, mostly above 90% ee) were achieved for different tetrahydroquinoline products **16** having *gem*-methyl ester groups (Scheme 12).

**Scheme 13:**
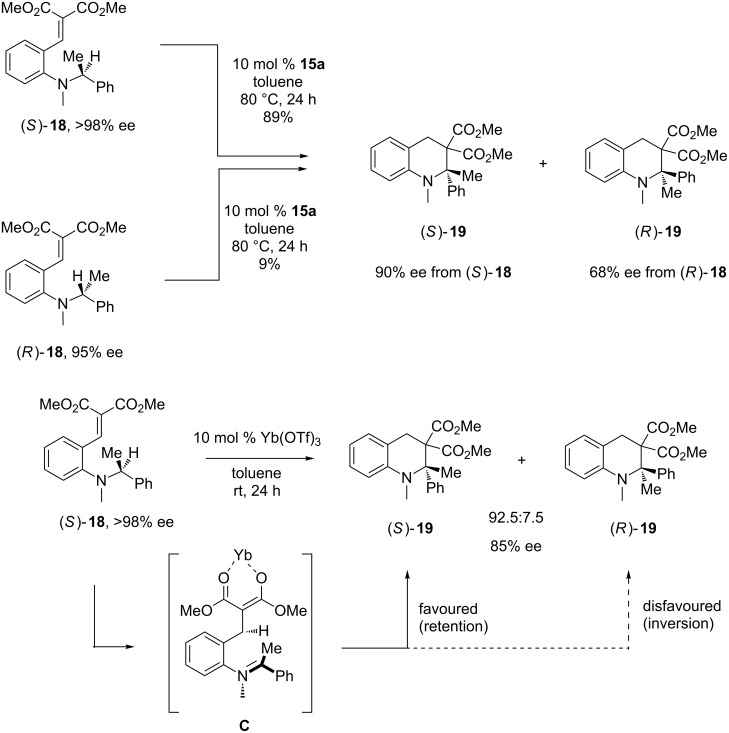
Asymmetric synthesis of tetrahydroquinolines from chiral substrates **18**.

For substrates containing one *N*-benzyl group and one *N*-ethyl group, binaphthol-based catalyst **17** was used; however, no chemoselectivity (**16j**:**16k** = 1.2:1) was observed (Scheme 12). Also, catalyst **15b** was used for the substrate having a *N,N*-diethyl group and product **16l** was obtained with lower enantioselectivity (70% ee). The authors carried out a series of model experiments with chiral substrates (*R*)-**18** and (*S*)-**18** to gain insight into the mechanism of their reaction (Scheme 13) [25].

In the presence of catalyst **15a**, (*S*)-**18** underwent a smooth reaction to provide product **19** with 90% ee in favour of the (*S*)-isomer. In contrast, when (*R*)-**18** was employed, the reaction was sluggish (10% yield) and only 68% ee of product **19** was observed in favour of the (*R*)-isomer. Even when achiral catalyst Yb(OTf)_3_ was used for the reaction with (*S*)-**18**, product **19** was obtained with 85% ee with the (*S*)-enantiomer as the major product. This clearly demonstrates that the chiral information in **18** did not disappear during the reaction and was retained as helical chirality in cationic intermediate **C** (Scheme 13). Nucleophilic attack then occurred from the same side of the transferred hydrogen to provide (*S*)-**19**. The authors concluded that selective activation of one of the enantiotopic hydrogen atoms by chiral phosphoric acid is the main reason for obtaining enantioselectivity for their reaction [25].

### Organocatalytic sp^2^ C–H bond activation reactions

**Scheme 14:**
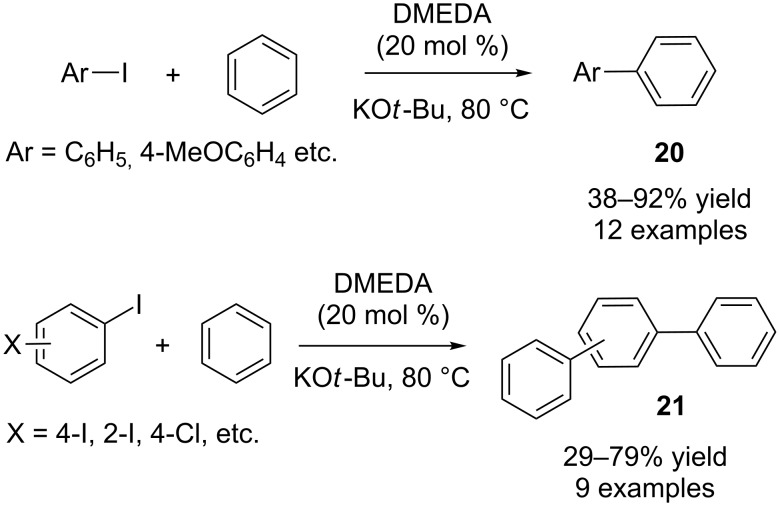
Organocatalytic biaryl synthesis by Kwong, Lei and co-workers.

The catalytic cross-coupling of arenes and aryl halides to construct biaryl compounds is an important area in synthetic organic chemistry. Transition-metal-catalyzed biaryl synthesis from unactivated arenes by C–H activation is well-known in the literature [26–30]. Stoichiometric amounts of a radical source, such as tributyltin hydride and tris(trimethylsilyl)silicon hydride [31], or irradiation [32] were also utilized for biaryl synthesis from unactivated arenes. However, organocatalysts have not been studied for this class of transformation. In 2010, three research groups independently reported organocatalytic biaryl synthesis from unactivated arenes and aryl halides [33–35]. Since these reactions follow a homolytic radical aromatic substitution mechanism (HAS) as pointed out by Studer and Curran [36], they are better termed as “organocatalytic direct arylations of arenes” rather than “C–H activation reactions”. Kwong, Lei and co-workers initially carried out the reaction between 4-iodotoluene and benzene with different bases and catalysts at 80 °C [33]. After varying bases and catalysts, potassium *tert*-butoxide (1.0 equiv) and DMEDA (*N*,*N*’-dimethylethane-1,2-diamine) were found to be the best base and catalyst, respectively, providing the desired product **20** in 84% yield (Scheme 14).

It is remarkable that *cis*-cyclohexane-1,2-diol is also a good catalyst for this reaction (81% yield). Under the optimized conditions, different aryl iodides were tested and good to excellent yields (38–92%) were obtained [33]. Dihalobenzenes were also employed as substrates, and poor to good yields (29–79%) for the products **21** were observed (Scheme 14). However, the reaction failed with anisole and toluene under the same reaction conditions.

**Scheme 15:**
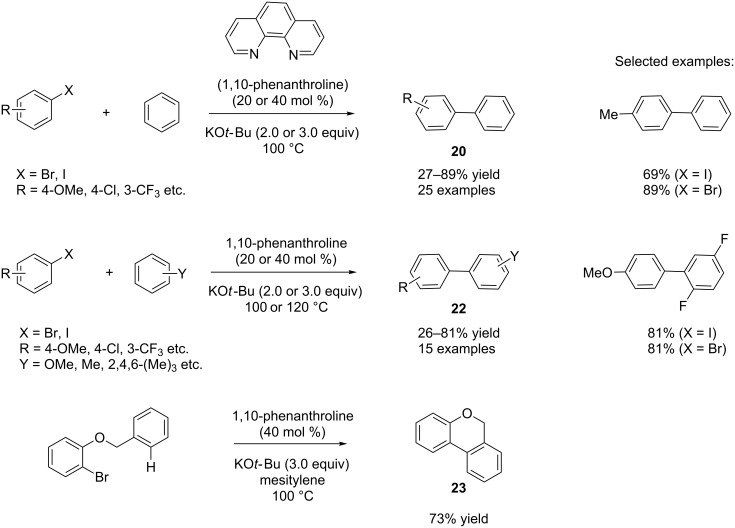
Organocatalytic biaryl synthesis by Shi and co-workers.

Shi and co-workers reported a similar reaction with 1,10-phenanthroline as catalyst at 100 °C employing aryl iodides and aryl bromides as the substrates [34]. Whereas 40 mol % of the catalyst and 3.0 equivalents of potassium *tert-*butoxide as base were needed for the reaction with bromides, 20 mol % of the catalyst and 2.0 equivalents of potassium *tert-*butoxide were required for the reaction with iodides. Under these optimized conditions, different aryl bromides and aryl iodides were screened, and poor to good yields (27–89%) for the products **20** were observed (Scheme 15) [34].

It is interesting that different arenes were also explored under the reaction conditions, and poor to good yields (26–81%) were attained for the desired products **22**. The authors found a decreased reactivity with increased electron density in the arenes; however, better conversion was obtained after long reaction time (2 days) at higher temperature (120 °C). The authors also discovered an intramolecular version of their reaction employing 1-(benzyloxy)-2-bromobenzene as the substrate in mesitylene as solvent, and 73% yield of the cyclized product **23** was obtained (Scheme 15).

**Scheme 16:**
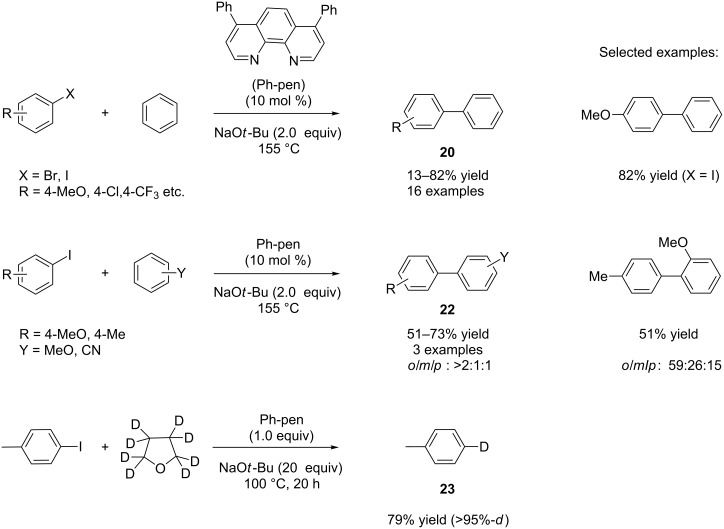
Organocatalytic biaryl synthesis by Hayashi and co-workers.

The third report of organocatalytic biaryl synthesis came from the group of Hayashi [35]. The combination of 4,7-diphenylphenanthroline (Ph-phen) as catalyst and sodium *tert*-butoxide as base at 155 °C was identified as the best system for their reaction. The authors applied their arylation method to different aryl and heteroaryl iodides as well as bromides, and poor to good yields (13–82%) for products **20** were obtained (Scheme 16) [35].

A variety of electron-donating and -withdrawing substituents were incorporated on the arene part and high *ortho*-selectivities were observed for the products **22**. The authors also investigated the reaction mechanism by performing a model reaction between 4-iodotoluene and THF-*d*_8_ with 20 equivalents of sodium *tert*-butoxide and Ph-phen (1 equiv) at 100 °C (Scheme 16). The formation of 4-deuterotoluene (**23**) implied the generation of a tolyl radical in the reaction, which finally abstracts a deuterium radical from THF-*d*_8_ to provide **23**. The authors found a low conversion (2%) for **23** in the absence of Ph-phen indicating the involvement of Ph-phen in the radical generation. The authors explained that Ph-phen can act as a single-electron-transfer (SET) mediator because it has a low lying LUMO and thus accepts an electron to generate a radical anion, and then passes the electron to aryl halide [35].

**Scheme 17:**
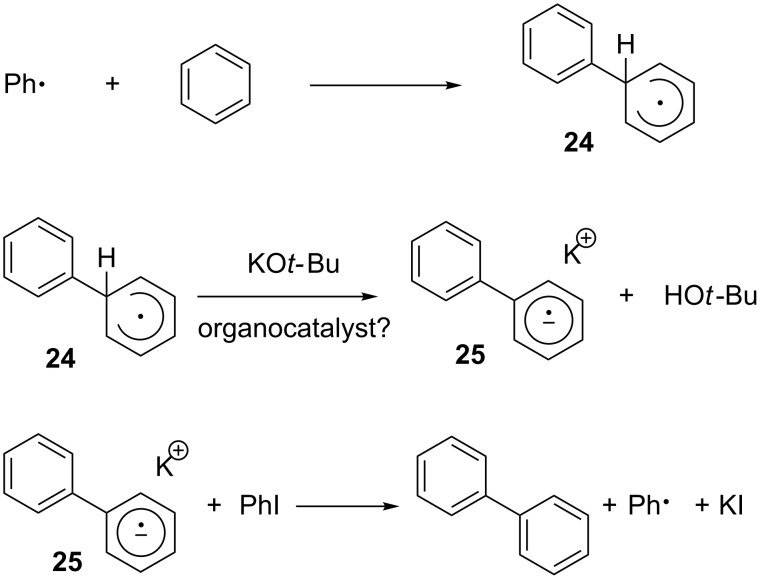
Proposed mechanism for organocatalytic biaryl synthesis.

A general mechanism for the organocatalytic cross-coupling reactions was proposed by Studer and Curran [36], which suggests a “base-promoted homolytic aromatic substitution” mechanism. In the first step, a phenyl radical generated from iodobenzene reacts with benzene to afford phenylcyclohexadienyl radical (**24)** (Scheme 17).

Radical **24** is then deprotonated by potassium *tert-*butoxide to generate the biphenyl radical anion (**25**), potentially promoted by an organocatalyst. In the last step, radical anion **25**, a strong reducing agent, transfers one electron to starting iodobenzene and results in the formation of biphenyl, potassium iodide and phenyl radical (Scheme 17). However, the role of the organocatalyst is still not fully understood at this point and detailed mechanistic studies are ongoing.

## Conclusion

In summary, this review highlights the recent developments of organocatalytic C–H activation reactions. Organocatalysts have been involved in 1,5-hydride shift and decarboxylative/non-decarboxylative redox-amination processes. Asymmetric organocatalytic C–H activation reactions have also been developed for the synthesis of chiral tetrahydroquinolines. Additionally, organocatalytic direct biaryl synthesis has been discovered; however, these are not considered to be “true” C–H activation reactions. It will be interesting to see true organocatalytic sp^2^ C–H activations in future, and more organocatalytic non-asymmetric and asymmetric sp^3^ C–H activation processes are expected [37].
